# Recent Advances in Toxic Wild Mushroom Distribution and Social Epidemiology

**DOI:** 10.3390/ijerph23040411

**Published:** 2026-03-25

**Authors:** Galina Yaneva, Tsonka Dimitrova, Djeni Cherneva, Ivelin Iliev, Kaloyan Mihalev, Svetlana Georgieva

**Affiliations:** 1 Department of Biology, Faculty of Pharmacy, Medical University of Varna, 9000 Varna, Bulgaria; tsonka.dimitrova@mu-varna.bg (T.D.); djeni_cherneva@abv.bg (D.C.); 2 Department of Pharmaceutical Chemistry, Faculty of Pharmacy, Medical University of Varna, 9000 Varna, Bulgaria; ivelin.iliev@mu-varna.bg (I.I.); kaloyan.mihalev@mu-varna.bg (K.M.); svetlana.georgieva@mu-varna.bg (S.G.)

**Keywords:** wild mushroom distribution, wild mushroom intoxications, epidemiology, morbidity, mortality

## Abstract

**Highlights:**

**Public health relevance—How does this work relate to a public health issue?**

- Wild mushroom consumption remains widespread globally and is frequently associated with severe foodborne intoxications due to misidentification of toxic species.
- This review provides updated data on the geographic distribution of poisonous mushrooms and the rising incidence of related intoxications in Asia, Europe, and the Americas.

**Public health significance—Why is this work of significance to public health?**

- Mushroom poisoning represents a leading cause of foodborne mortality in several regions, particularly due to amatoxin-containing species such as *Amanita phalloides* and *Amanita exitialis*.
- The synthesis of newly identified toxic species and evolving epidemiological patterns contributes to improved understanding of current morbidity and mortality risks.

**Public health implications—What are the key implications or messages for practitioners, policy makers and/or researchers in public health?**

- Mapping regional toxicity patterns supports clinical toxicologists and emergency physicians in improving diagnostic accuracy and timely treatment.
- Identification of seasonal peaks and high-risk demographic groups provides evidence for targeted public health education campaigns and preventive food safety policies.

**Abstract:**

Wild mushroom consumption is widespread worldwide and remains an important cause of foodborne intoxication. This concise review analyzes recent literature on the geographic distribution of poisonous wild mushrooms and the epidemiological patterns of intoxication reported in Asia, Europe, and the Americas. Most poisoning incidents occur as a result of the misidentification of toxic species as edible during mushroom foraging. Alongside well-known poisonous mushrooms, several newly identified toxic species have been reported in recent years. The available epidemiological evidence demonstrates clear regional clustering of poisoning incidents, pronounced seasonal peaks associated with mushroom growth, and a predominance of cases in populations where wild mushroom foraging is a traditional practice. Amatoxin-containing species of the genus *Amanita* remain the leading cause of severe and fatal intoxications worldwide. Overall, the analyzed studies indicate that wild mushroom poisoning continues to represent a significant food safety and public health concern, particularly in Asia and parts of Europe. Improved toxicological surveillance, public awareness, and timely clinical management are essential for reducing morbidity and mortality associated with these intoxications.

## 1. Introduction

Nowadays, there is a rising scientific interest in the socially significant field of poisonous wild mushrooms worldwide. They are widely distributed and ingested. The consumption of certain wild mushrooms by the populations in many countries is sometimes associated with severe morbidity and even mortality in adults and children. Wild mushroom intoxication presents with a variety of symptoms and can cause organ failure, necessitating timely diagnosis and proper treatment.

The purpose of this concise review is to discuss the recent literature dealing with some aspects of the geographic distribution and social epidemiology of toxic wild mushrooms.

## 2. Geographic Distribution of Poisonous Wild Mushrooms

The results from a bibliometric analysis of a total of 985 publications (each with a minimum of five citations) dealing with mushroom poisoning identified in the *Scopus* database indicate that one-third of these articles are published in 31 ‘core’ journals [1]. Leading the list is *Clinical Toxicology* (*Philadelphia*) with 41 papers. Authors from the USA contribute to 19.6% of all the publications on this topic. China is an emerging leader in research output since 2011. *Amanita phalloides*-related research is the most frequently published topic.

Nephrotoxic mushroom intoxication has been described in several studies investigating its epidemiology, clinical manifestations, and management. A comprehensive review of the literature published since 1957 identifies several mushroom species capable of causing acute renal toxicity [2]. A toxidromic approach to the early diagnosis of these poisonings based on the onset of acute renal failure is presented, and the outcomes of renal replacement management strategies, including hemodialysis and renal transplantation in cases of failed extracorporeal treatment, are compared. Most commonly, *Cortinarius* species can cause acute renal insufficiency. The newly identified species of nephrotoxic mushrooms are *Amanita proxima* and *Tricholoma equestre* in Europe, and *Amanita smithiana* in the United States and Canada.

Global diversity and distribution patterns of ectomycorrhizal fungi are addressed with a focus on *Amanita* section *Vaginatae* of African origin [3]. Major biogeographic ‘out-of-Africa’ events include multiple dispersal events to Southeastern Asia, Madagascar, and the current Amazonian basin—the last two likely being transoceanic. Later events originating in Southeastern Asia involve Nearctic dispersal to North America, Oceania (Australia and New Zealand), and Europe. Subsequent dispersals are also inferred from Southeastern to Eastern Asia; from North America to Eastern Asia, Southeastern Asia, Northern Andes and Europe; and from the Amazon to the Caribbean region.

A list of poisonous mushrooms, including 643 species from two phyla, 16 orders, 51 families, and 148 genera, has been compiled, of which 332 species have been toxicologically studied and associated with documented clinical poisoning cases, while 311 species have previously been reported as poisonous in other investigations [4].

A summary of representative poisonous wild mushroom species reported in the literature, together with their associated clinical manifestations, is presented in Table 1.

*Gymnopilus junonius* is a globally distributed toxic mushroom [25], notable for containing psilocybin—a hallucinogenic alkaloid—alongside various other bioactive substances. Numerous toxic *Amanita* species similarly exhibit worldwide distribution and pose significant public health concerns, contributing to both morbidity and mortality [27]. To date, more than 16,000 mushroom species have been documented globally, of which over 4000 occur in China; among these, upwards of 400 are classified as toxic [28]. Wild mushroom foraging holds deep cultural roots in many Chinese regions, especially in mountainous provinces such as Sichuan, Yunnan, and Guizhou [29,30]. Chlorophyllum molybdites represents another frequently encountered poisonous species in China, with a broad geographic range across the country [18]. Two novel species belonging to the genus Inosperma (family Inocybaceae)—namely Inosperma muscarium and Inosperma hainanense—have been described from tropical China on the basis of morphological characteristics and multilocus phylogenetic analysis, with both demonstrating unexpectedly high muscarine content [26].

Among wild mushrooms, *Amanita muscaria* stands out for its immediately recognizable morphology, making it one of the most visually distinctive mushrooms known [23]. The species has a broad global range and is a familiar sight across continental Europe and the United Kingdom, typically fruiting between July and October. In both the Americas and Europe, the most dangerous and frequently lethal representatives of the genus are *Amanita phalloides*, *Amanita verna*, and *Amanita virosa* [17]. The mushroom diversity of Israel is notably rich in toxic taxa, with approximately 65 species classified as poisonous or potentially hazardous within the country’s mycobiota [31].

Two cases of severe *A. muscaria* intoxication have been documented in Trabzon, Turkey [24]. The first case involves a 44-year-old male patient who was admitted to the emergency department following cardiopulmonary arrest occurring approximately ten hours after the ingestion of four to five dried *A. muscaria* caps. The second case concerns a 75-year-old male who presented to the emergency department following the accidental consumption of a single large A. muscaria cap that had been self-foraged in Eastern Turkey.

A comprehensive multidisciplinary reassessment of European *Amanita* species belonging to section *Phalloideae*, incorporating morphological, phylogenetic, epidemiological, and toxicochemical data on amatoxins and phallotoxins, has delineated five recognized species within Europe [32]. These comprise *A. phalloides*, *A. virosa*, and *A. verna*, alongside two more recently introduced North American taxa: *A. amerivirosa* and the newly described *A. vidua* sp. *nov*. Furthermore, three previously recognized taxa—*A. decipiens*, *A. porrinensis*, and *A. virosa* var. *levipes*—have been reclassified as heterotypic synonyms of *A. verna*, *A. phalloides*, and *A. amerivirosa*, respectively.

Within Europe, the highest frequency of mushroom poisoning cases has been recorded in Eastern Europe, with notable concentrations in the coniferous forest regions of Germany, Poland, and Finland [22]. In North America, Michigan accounts for the greatest number of reported exposures, while a comparatively less toxic variety is predominantly distributed west of the Rocky Mountains, with case clusters documented in Idaho and Western Canada. In contrast to other severe poisoning syndromes, such as those associated with *A. phalloides*, which exhibit an autumn predominance, *Gyromitra*-related intoxications occur most frequently during spring months.

Historically, poisonings attributable to *Gyromitra* consumption have been documented across Europe and the United States over several centuries, with taxonomic reclassifications complicating retrospective attribution. Early recorded cases in France in 1793 were initially linked to a mushroom which was then designated as *Morchella pleopus*. Subsequent phytochemical investigations led to the characterization of an extract termed ‘helvellic acid’ in 1885, while the causative toxin gyromitrin was not isolated and structurally elucidated until 1968, when List and Luft accomplished this in Germany. Epidemiological records from Poland document 138 intoxications and two fatalities between 1953 and 1962. The Swedish Poison Information Center registered 706 inquiries concerning *Gyromitra* species between 1994 and 2002, with no associated fatalities. In the United States, poison centers recorded 82,140 mushroom-related calls between 2001 and 2011, of which 448 involved gyromitrin-containing species. Over a thirty-year surveillance period, the North American Mycological Society documented 27 confirmed cases of Gyromitra intoxication [22].

## 3. Results

### 3.1. Morbidity of Wild Poisonous Mushrooms

The scientific literature on mushroom intoxication extends back to at least 1837, the date of the earliest identified publication on the subject [1]. The predominant mechanism underlying poisoning incidents is the misidentification of toxic species as edible counterparts by amateur foragers [33].

Rising global mushroom consumption, combined with deeply rooted cultural traditions of wild mushroom foraging prevalent in Romania and across Eastern Europe more broadly, has been associated with an increasing number of emergency department presentations attributable to the ingestion of inedible species [34]. In France, approximately 1300 cases of wild mushroom intoxication are recorded annually, with fatal outcomes predominantly linked to *A. phalloides* [5]. In Turkey, *A. phalloides* has similarly been identified as the principal causative agent of severe mushroom poisoning, a finding attributed both to its widespread distribution and to the considerable toxic potency of its amatoxin content per unit mass [6]. Surveillance data from Slovakia covering the period from 2004 to 2020 document a cumulative total of 2876 registered cases of wild mushroom poisoning [35], among which 698 were classified as suspected *A. phalloides* intoxications and 141 were laboratory-confirmed.

Epidemiological data from the Israel Poison Information Center in Haifa offer valuable insights into the patterns of wild mushroom intoxication at the national level. A retrospective review of the period from 2015 to 2020 recorded 105 calls related to mushroom consumption in 2020 alone [15], of which 65 cases (61.90%) were registered during the final quarter of the year. This figure represented a 2.5-fold increase relative to the median annual rate documented between 2015 and 2019, and a five-fold increase when compared to the corresponding autumn-winter period of 2019. Moderate to severe intoxications, including one life-threatening case, were identified in 6% of patients, with *Lepiota brunneoincarnata* and *A. proxima* being the principal species implicated. A separate retrospective analysis from the same center encompassing the years 2010 to 2021 established that wild mushrooms accounted for approximately 4% of all exposures to biological agents [31]. A statistically significant male predominance was observed. The age groups most frequently represented were adults over 18 years and children under six years of age, comprising 41% and 39% of cases, respectively. Between 2017 and 2021, a total of 128 incidents involving the consumption of raw wild mushrooms were recorded, with the majority occurring in children below six years of age.

Analysis of the Healthcare Cost and Utilization Project database reveals that in 2016, accidental ingestion of poisonous wild mushrooms was associated with an estimated 1328 ± 100 emergency department visits and 100 ± 22 hospitalizations across the United States [36]. The patient cohort comprised 832 males and 496 females. The most substantially represented age groups were individuals between one and 17 years of age (548 cases) and those between 18 and 44 years (511 cases), with an additional 180 patients falling within the 45 to 64 year age bracket.

A retrospective study conducted in Elazig, Turkey, identified a total of 143 children between one month and 18 years of age who were hospitalized for intoxication during the period from January 2015 to October 2017 [37]. Among the 19 children presenting specifically with toxic plant or mushroom poisonings, seven cases (36.84%) were attributable to wild mushroom ingestion. A seasonal predisposition was observed, with the majority of such intoxications occurring during the spring months.

In Japan, a total of 86 poisoning incidents involving 347 individual patients attributed to *Tricholoma ustale* have been documented over the period from 1989 to 2010 [21].

Data from a tertiary care institution in North-Eastern India indicate that 44 patients with a mean age of 20.13 ± 15.39 years were admitted for wild mushroom poisoning between January 2015 and December 2020 [38]. The cohort consisted of 23 male and 21 female patients. With respect to age distribution, 17 patients were between 19 and 60 years of age, 15 fell within the two to 12 year range, and the remaining 12 were between 13 and 18 years old.

A descriptive retrospective cross-sectional study conducted at Razi Hospital in Qaemshahr, Mazandaran, Iran, recorded 65 patients hospitalized for wild mushroom poisoning between 2015 and 2018 [39]. The cohort had a mean age of 35.68 years and comprised 33 males (mean age 35.93 years) and 32 females (mean age 35.43 years), with 33 patients belonging to the age group of 31 years or younger. A pronounced seasonal distribution was observed: spring accounted for the greatest proportion of cases (39 patients; 60.00%), followed by autumn (18 patients; 27.69%), summer (five patients; 7.69%), and winter (three patients; 4.62%).

A hospital-based cohort from Kermanshah province, Western Iran, encompassing 193 patients with wild mushroom poisoning admitted between March 2014 and March 2018, reported a mean patient age of 43.1 ± 16.2 years [16]. The sex distribution was nearly equal, with 99 males (51.30%) and 94 females (48.70%). The predominant age groups were those between 21 and 40 years (72 cases) and between 41 and 60 years (68 cases), collectively accounting for the majority of the study population.

An outbreak of poisoning associated with cyclopeptide-containing wild mushrooms was reported in the same province in 2018, involving a total of 283 affected individuals [40]. The cohort comprised 143 males and 140 females. Approximately 43% of cases were managed on an outpatient basis, while around 40% of patients required hospitalization within one to three days of exposure. Age distribution analysis revealed that the largest subgroup consisted of individuals between 20 and 39 years of age (133 patients), followed by those aged 40 to 59 years (98 patients), 13 to 19 years (28 patients), and patients aged 60 years or older (24 patients).

Between 2003 and 2017, there are a total of 22,571 cases of wild mushroom intoxications associated with the rainfall in Thailand [41]. The yearly cases range from 1232 in 2014 (1.9 per 100,000 population) to 2148 in 2012 (3.3 per 100,000 population). There are between nine cases in April 2016 and February 2017, and 438 cases in May 2012. There is a domination of females over males (13,326 versus 9245) and mature adults (aged 35–44 years) over children (aged ≤ 14 years) or elderly persons (aged ≥ 65 years). The patients living in rural areas are statistically significantly more than those living in urban areas (*p* = 0.003). The cases in the northeastern and northern regions are more common than those in the central, south, and east regions of the country. There are 17,337 poisoned patients in the wet season (from May to September) and 5234 in the dry season (between October and April). A strong positive correlation between the number of monthly poisoning cases and the amount of monthly rainfall is established (Spearman’s correlation coefficient r_s_ = 0.801; *p* < 0.001).

Within a retrospective cohort study of myotoxic mushroom intoxications during the period between January 2012 and December 2016, there are 41 patients, 22 males and 19 females, at a mean age of 48.85 ± 16.02 years (range: 15 to 79 years) registered in the Ramathibodi Poison Center Toxic Exposure Surveillance System in Thailand [20]. *Russula* species are identified in three patients. Within the first 24 h after mushroom ingestion, 29 patients (70.73% of the cases) are hospitalized. The median time to admission after mushroom consumption is 21 h (range: 2 to 120 h).

Mushroom poisoning poses a significant food safety concern in China, with a total of 196 species identified in poisoning incidents by the end of 2022 [9]. A total of 97 mushrooms are identified as the cause of six distinct clinical disease types, with 12 species newly documented as poisonous mushrooms in the country.

Epidemiological studies from China indicate that wild mushroom poisoning represents a significant public health concern characterized by clear seasonal patterns, regional clustering, and the involvement of several highly toxic species. Amatoxin-containing mushrooms, particularly species of the genus *Amanita*, are among the most dangerous causes of fatal intoxications, while other species, such as *Chlorophyllum molybdites* are frequently involved in non-fatal outbreaks [19]. For example, in 2019 alone, 55 food intoxication incidents in China are attributed to accidental consumption of *Chlorophyllum molybdites* [18]. In addition, several poisoning events caused by the highly lethal species *A. exitialis* have been reported in southern China. A retrospective analysis of ten poisoning events involving 27 individuals in Chuxiong Yi Autonomous Prefecture between 2019 and 2024 reveals a mean patient age of 46.8 ± 22.1 years, with nearly equal sex distribution (14 females and 13 males) and a latency period of approximately 12.0 ± 5.0 h, the time from ingestion to hospital admission is 45.9 ± 8.6 h, and the hospital stay is 8.2 ± 5.8 days [10].

National surveillance data further illustrate the scale of this problem. Analysis of the Public Health Emergency Management Information System of the China CDC shows that between 2012 and 2023 only 14.08% of poisoning incidents included laboratory-confirmed identification of the responsible species, with *A. exitialis* and *Russula subnigricans* accounting for the largest proportion of cases (17.39%) [11]. Between 2010 and 2020, a total of 10,036 mushroom poisoning outbreaks are reported in China, resulting in 38,676 poisoning cases and 788 deaths [42,43]. These incidents occur throughout the country but are most common in the southwestern and central regions. Most outbreaks are associated with food prepared in households (84.6%), followed by street food consumption (8.7%) and institutional food settings (2.5%).

A pronounced seasonal pattern is also observed. The peak period of mushroom poisoning occurs from summer to autumn, particularly between May and October, accounting for 94.1% of outbreaks and 97.2% of lethal cases [43]. Additional regional studies support these findings. For example, between 2016 and 2018, 429 cases of wild mushroom intoxication were reported from 340 sentinel hospitals in Zhejiang Province, with an incidence rate of 0.2526 per 100,000 population [42]. Surveillance investigations conducted by the China CDC also report 276 poisoning incidents involving 769 patients in 2019 and 327 incidents involving 923 patients in 2021 across multiple provincial administrative divisions [44,45].

These investigations identify numerous toxic species responsible for several clinical syndromes. Between 2015 and 2020, a total of 4841 patients with wild mushroom intoxication are hospitalized in 17 hospitals in Chuxiong Prefecture, China [46]. Among them, 2776 patients (57.34% of the cases) have information recorded concerning the identification of the mushroom species involved. In 2022, 482 mushroom poisoning incidents involving 1332 patients are recorded across 21 provincial-level administrative divisions, with 98 poisonous species identified as causes of seven distinct clinical syndromes [12]. In addition to well-known toxic species, several newly described mushrooms such as *Collybia humida*, *Spodocybe venenata*, and *Omphalotus yunnanensis* are recently recognized as poisonous in China [12]. Overall, surveillance data indicate that mushroom poisoning remains one of the most frequent causes of foodborne outbreaks in the country. In 2020 alone, 2705 outbreaks of wild mushroom intoxication are reported among 4662 foodborne outbreaks with confirmed etiology, accounting for 58.02% of all outbreaks and affecting 9111 patients [47].

Overall, the epidemiological data presented above reveal several consistent global trends in wild mushroom poisoning. The most dangerous species implicated in fatal outcomes are amatoxin-containing mushrooms, particularly *Amanita phalloides* and *Amanita exitialis*, with *Lepiota* and *Russula* species also contributing to severe intoxications across multiple regions. Regarding affected populations, adult males appear disproportionately represented in most cohorts, although children under six years of age constitute a vulnerable subgroup, especially in cases of accidental ingestion of raw mushrooms. A clear seasonal pattern emerges across geographically diverse settings: poisoning incidents peak during spring and summer-to-autumn months, corresponding to periods of active mushroom growth, a trend particularly pronounced in Asia, where rainfall strongly correlates with incidence. Geographically, rural populations and those in regions with strong traditions of wild mushroom foraging, including Eastern Europe, Western and Central Asia, and southwestern China, bear the greatest burden of disease. Taken together, these patterns underscore the need for targeted public health interventions during peak foraging seasons and among high-risk communities.

### 3.2. Mortality of Wild Poisonous Mushrooms

Wild mushroom poisoning constitutes an enduring public health challenge associated with considerable mortality, with the burden of disease disproportionately concentrated in populations where the foraging of wild fungi represents an established cultural tradition [48]. Within the spectrum of toxic species, Amanita phalloides is widely recognized as one of the most dangerous and potentially fatal mushrooms encountered globally [6].

In Europe, approximately between 50 and 100 fatal cases due to mushroom poisoning are reported each year [7]. In the USA, wild and potentially toxic mushroom consumption is a common practice. During the period between 1999 and 2016, there were 704 severe intoxications alone, with 52 fatalities among a total of 133,700 poisoning cases, mainly resulting from cyclopeptide-containing mushrooms in adults.

Diagnosis-related group data from Germany for the period 2000 to 2018 document a cumulative total of 4412 hospitalizations and 22 fatalities resulting from toxic mushroom consumption [8], with approximately 90% of lethal outcomes attributable to *A. phalloides* intoxication.

A multicentre nationwide retrospective cohort analysis drawing on the National Electronic Database of the Turkish Ministry of Health identified 30,459 individuals-16,829 women and 13,630 men-with a mean age of 45.8 ± 20.3 years (range: 4 to 74 years) who were admitted for mushroom intoxication between January 2018 and December 2023 [6]. Among hospitalized patients, the 30-day and 90-day mortality rates were 4.38% (119 cases) and 6.56% (178 cases), respectively. A statistically significant age difference was observed between deceased patients and survivors, indicating that older age constitutes a significant risk factor for fatal outcomes.

The results from the retrospective investigation of the mortality rate of suspected cyclopeptide-containing mushroom consumptions reported to the National Poison Data System of the USA, during the period between January 2008 and December 2018, display a total of 8953 wild mushroom exposures [49]. There are 13 lethal cases among 148 patients, showing a total mortality rate of 8.78%. The mortality rate is 9.52% in 42 silibinin/silymarin-treated patients and 8.49% in 106 untreated ones. Wild mushrooms are identified in 16.89% of the cases, as 80.00% of them contain cyclopeptides. Among these confirmed cases, the mortality rate is 10.00% in both silibinin/silymarin-treated and untreated patients.

The mortality rate of 193 patients with *A. virosa* mushroom poisoning in Kermanshah province, Western Iran, is 1.55% (three lethal cases) [16]. During the period between January 2015 and December 2020, there are ten lethal cases among a total of 44 patients with wild mushroom intoxication in Meghalaya, North-Eastern India [38]. This in-hospital mortality rate amounts to 22.73%.

Amatoxin intoxication represents the main cause of death due to accidental wild poisonous mushroom consumption in Thailand, with a mortality rate of 27.3% [50].

During the period between 2003 and 2017, a total of 106 deaths caused by wild mushroom poisoning are registered in Thailand [41]. There was only one lethal case in 2005 and 2006, respectively; however, there were 24 deaths in 2012 (0.04 per 100,000 population). Most lethal cases (19) are registered in May 2012.

The results from a retrospective cohort study of myotoxic mushroom intoxications during the period between January 2012 and December 2016 in Thailand show 11 lethal cases (26.82%) among 41 patients (22 males and 19 females) at a mean age of 48.85 ± 16.02 years (range: 15 to 79 years) [20].

In China, fatal mushroom poisoning is predominantly attributable to a distinct group of highly toxic species, namely *A. exitialis*, *A. subjunquillea*, *A. pseudoporphyria*, *A. subpallidorosea*, and *A. rimosa* [9,12]. Of these, *A. exitialis* currently represents the most lethal species associated with mushroom poisoning incidents in the country. Surveillance data covering the period from 2019 to 2023 recorded 135 cases and 24 deaths linked to *A. exitialis* poisoning [9,12]. A family outbreak in Yunnan Province in 2019 involving five individuals resulted in the death of one of two children who had ingested *A. exitialis* [13]. A subsequent incident in Shenzhen, Southern China, in April 2022, documented one fatality among ten patients presenting with *A. exitialis* intoxication [14].

Among 27 patients (14 females and 13 males) at a mean age of 46.8 ± 22.1 years poisoned by *A. exitialis* between 2019 and 2024 in Chuxiong Yi Autonomous Prefecture People’s Hospital, China, there were five deaths (18.52% of the cases) [10].

According to the Public Health Emergency Management Information System, China CDC, most lethal outcomes of mushroom poisoning during the period from 2012 to 2023 are due to acute liver failure (55.17% of the cases) [11]. *A. exitialis* and *A. fuliginea* cause most intoxications and deaths, accounting for 13.32% and 18.10% of the cases, respectively.

The comparison between the mean mortality rates of wild mushroom intoxications between 2015 and 2017 and those between 2018 and 2020 in 17 hospitals in Chuxiong Prefecture, China, reveals the statistically significant diminution of these rates from 0.57% down to 0.06% (*p* < 0.001) [46].

Epidemiological data indicate that mushroom poisoning is now one of the leading causes of food-borne disease outbreaks and mortality in China [51,52]. During the period from 2010 to 2020, a total of 10,036 mushroom poisoning outbreaks were reported in the country, resulting in 788 deaths [47]. In 2020 alone, 80 lethal cases due to wild mushroom poisoning were recorded, representing 55.94% of all foodborne outbreak-related deaths in China and corresponding to a case fatality rate of 0.9% [47].

The overall mortality rate due to wild mushroom intoxications assessed by the Chinese Center for Disease Control and Prevention in 17 provincial-level administrative divisions in China is 2.86% (22 out of a total of 769 poisoned patients) in 2019 [44]; in 25 provincial-level administrative divisions, it is 2.17% (20 out of a total of 923 intoxicated patients) in 2021 [45]; and in 21 provincial-level administrative divisions, it is 2.10% (28 out of a total of 1332 poisoned patients) in 2022 [12]. In 2023, an investigation of 505 cases of mushroom poisoning across 24 provincial-level administrative divisions revealed a total of 303 patients, 16 deaths, and a case fatality rate of 1.23% [9].

During the period between July 2007 and August 2017, a total of 66 patients with acute wild mushroom intoxication admitted to the First Affiliated Hospital of Dalian Medical University in Dalian, China, were retrospectively studied [53]. There are 44 patients with liver injury and 22 patients with liver failure. Ten out of the 22 patients with liver failure die, as the mortality rate in this group amounts to 45.45%.

In Zhejiang province, China, between January 2016 and December 2018, there were two lethal cases in 2016 only, due to wild mushroom poisoning among a total of 429 patients, accounting for a mortality rate of 0.47% [42].

Three patients—one 12-year-old girl, her 61-year-old grandmother, and her 63-year-old grandfather—were hospitalized in the emergency department due to intoxication with wild mushrooms picked from a nearby forest area in India, are reported [54]. After combined treatment; however, all three patients died.

Twelve hours after *Amanita* mushroom consumption, a 54-year-old woman, her daughter, and her son-in-law are admitted to the emergency department with a presumed diagnosis of food poisoning in Columbia, USA [55]. The female patient died four days after mushroom ingestion, despite intensive complex treatment.

A summary of representative epidemiological studies on wild mushroom poisoning and associated mortality worldwide is presented in Table 2.

Overall, available epidemiological evidence indicates that China represents one of the regions most heavily affected by fatal wild mushroom intoxications worldwide. National surveillance data demonstrate that mushroom poisoning accounts for a substantial proportion of food-borne disease outbreaks and associated mortality in the country. The majority of lethal cases are linked to amatoxin-containing species of the genus *Amanita*, particularly *A. exitialis*, *A. subjunquillea*, *A. pseudoporphyria*, *A. subpallidorosea*, and *A. rimosa*. Acute liver failure remains the principal cause of death in these poisonings. Although national surveillance programs and improved clinical management have contributed to a gradual decline in mortality rates in recent years, mushroom intoxication continues to represent a significant public health concern, particularly in rural areas where wild mushroom foraging is a common practice. These findings highlight the importance of continuous toxicological surveillance, public education, and rapid clinical intervention in reducing mortality associated with poisonous wild mushrooms.

## 4. Discussion

The studies summarized in this review collectively indicate that wild mushroom intoxication is not merely an accidental toxicological phenomenon but a predictable outcome of the interaction between environmental availability, cultural practices, and the limits of species recognition outside expert mycology. A consistent driver across settings is the misidentification of toxic species as edible, which is an error that becomes more likely when mushroom foraging is widespread, informal, and transmitted through non-standardized folk knowledge. The observed clustering of cases in regions where foraging is a cultural tradition (e.g., Eastern Europe and several Asian regions) is therefore not surprising, and it reflects higher exposure probability rather than a unique biological property of the local fungi. In this context, poisoning incidence functions as an indirect indicator of social behavior, access to expert identification, and the availability of preventive infrastructures (poison centers, surveillance systems, and educational campaigns), which vary substantially between countries.

An integrative trend is the recurrent dominance of amatoxin-associated severe outcomes, especially in regions with high exposure to toxic *Amanita* species. This pattern has a mechanistic and clinical explanation: amatoxin intoxication typically manifests after a latency period, which may delay clinical recognition and hospital presentation. Such delays, explicitly captured in hospital-based cohorts, create a narrow window for optimal intervention and increase the risk of acute liver failure, which appears as the principal fatal pathway in large surveillance datasets. In contrast, the high frequency of non-fatal outbreaks linked to species such as *Chlorophyllum molybdites* can be interpreted as a “high-incidence/low-fatality” model, where widespread exposure results in large numbers of gastrointestinal cases that burden emergency services but contribute less to mortality. This distinction is important for public health planning because it implies that morbidity and mortality are driven by partially different “species–syndrome” constellations, and therefore require different prevention and triage priorities.

When linking subthemes across the morbidity and mortality sections, several meaningful consistencies and some apparent tensions emerge. There is a broad consensus on seasonality: most regions show peaks during periods of maximal mushroom growth and active foraging. However, the season of highest risk is not uniform; it is shaped by climate, rainfall patterns, and local species phenology. For example, datasets from Thailand explicitly demonstrate a strong association with rainfall and a clear wet-season burden, suggesting that ecological drivers may dominate there, while in China and other temperate regions, the peak period tends to concentrate from late spring/summer through autumn. These differences are not contradictions but reflections of different ecological contexts, and they underline why prevention messaging must be region-specific rather than global and generic.

Demographic patterns across studies also show both consensus and heterogeneity. Children appear repeatedly in exposure datasets, likely reflecting exploratory ingestion, accidental tasting, and caregiver-related risk factors rather than intentional consumption. Meanwhile, adults, especially those actively foraging, dominate intentional ingestion cases and may experience more severe outcomes because of higher ingested doses and delayed care-seeking. Yet, cross-country comparisons are limited by differences in study design: some datasets are hospital-based, others are poison-center based, and some derive from national surveillance systems. These sources capture different clinical spectra: poison centers may be enriched for milder cases and inquiries, hospitals for more severe presentations, and surveillance systems for outbreaks and clusters. Therefore, differences in age distributions between countries may reflect, at least in part, differences in surveillance architecture rather than true epidemiologic divergence.

From a critical perspective, the quality of evidence across the reviewed literature is uneven, and this review exposes several knowledge gaps that limit inference. First, a major limitation is the frequent absence of species-level confirmation. Even in large national systems, laboratory-confirmed identification constitutes a minority of incidents, which constrains robust attribution of syndromes, severity, and fatality to specific taxa. This gap has downstream consequences: without reliable identification, risk estimates for individual species, regional risk mapping, and targeted prevention strategies remain uncertain. Second, many studies are retrospective, rely on secondary databases, and use inconsistent case definitions (e.g., “mushroom poisoning” as a broad category without standardized clinical criteria). Third, reporting biases are likely substantial; cases that do not seek care, mild intoxications treated at home, and exposures not reported to poison centers remain invisible, while outbreaks and severe cases are overrepresented. Finally, clinical outcome reporting is heterogeneous: some studies provide detailed latency and hospitalization metrics, whereas others focus on aggregate counts, limiting meta-level comparisons.

Despite these constraints, the review provides a theoretical and practical contribution by reframing wild mushroom intoxication as a preventable, systems-level problem rather than an isolated individual error. Theoretically, the converging evidence supports a model in which the burden of poisoning is determined by (i) exposure intensity (foraging prevalence), (ii) ecological opportunity (seasonality, rainfall, habitat), (iii) availability of highly toxic species (notably amatoxin-containing Amanita), and (iv) response capacity (surveillance, early recognition, access to specialized care). Practically, this implies that prevention should be stratified: regions with frequent gastrointestinal outbreaks may benefit most from broad public education and consumer guidance (including warnings about market purchases, mixed mushrooms, and raw consumption), while regions with documented amatoxin-related mortality require high-priority messaging on delayed toxicity, early hospital evaluation, and strengthened clinical pathways for suspected amatoxin exposure. Moreover, the demonstrated contribution of household-prepared foods to outbreaks in some national datasets suggests that prevention cannot rely only on commercial food safety regulation; it must also address informal collection and home preparation.

An additional practical implication concerns surveillance and comparability. Because evidence quality is strongly shaped by whether countries have poison centers, systematic reporting, and laboratory confirmation capacity, improving surveillance is not merely an academic exercise, but it directly improves public health decision-making. Strengthening diagnostic mycology support (morphological and molecular identification where feasible), standardizing reporting templates (including minimum datasets such as age, setting, season, latency, suspected species, and outcome), and integrating poison center data with hospital outcomes would enable higher-resolution risk assessment. These measures would also facilitate the production of comparable datasets across countries.

Finally, beyond summarizing what is known, the review supports a more critical stance toward the persistent societal normalization of wild mushroom consumption by non-experts. The recurring pattern of severe outcomes due to misidentification, the presence of newly documented poisonous species, and the continued burden on emergency care systems collectively suggest that risk communication should be explicit and unambiguous: non-expert foraging and consumption are inherently unsafe, particularly during peak seasons and in regions where highly toxic species are present. From the authors’ perspective, the literature does not justify complacency or the assumption that traditional knowledge alone is sufficient for safe consumption. Instead, the evidence argues for prevention strategies that combine culturally sensitive communication with clear warnings, strengthened surveillance, and clinical readiness, especially during high-risk seasons and in regions with documented amatoxin-associated mortality.

## 5. Conclusions

The present review highlights the growing scientific interest in the global distribution and epidemiology of poisonous wild mushrooms and confirms that mushroom intoxication remains a significant public health concern worldwide. Numerous toxic species have been reported in the literature, with particular attention given to highly dangerous mushrooms such as *A. phalloides*, *A. exitialis*, *A. virosa*, and other amatoxin-containing species, which are responsible for the majority of severe and fatal poisonings. Other species, including *Chlorophyllum molybdites*, *Russula* spp., and *Tricholoma* species, are frequently associated with gastrointestinal or myotoxic syndromes and contribute substantially to the overall morbidity related to wild mushroom consumption.

The reviewed epidemiological studies demonstrate clear geographic and seasonal patterns. Severe poisonings are most frequently reported in Asia and parts of Europe, particularly in regions where wild mushroom foraging is a traditional cultural practice. Seasonal peaks are commonly observed during the summer and autumn months, when environmental conditions favor mushroom growth and collection. In many countries, the majority of intoxications occur as a result of the misidentification of poisonous species as edible mushrooms by amateur foragers.

The potential health risks associated with the consumption of wild mushrooms are considerable and may include severe gastrointestinal toxicity, acute renal injury, and acute liver failure, which remains the leading cause of death in cases involving amatoxin-containing species. Despite improvements in clinical management and toxicological surveillance, mushroom poisoning continues to cause substantial morbidity and mortality in several regions of the world.

From a public health perspective, the findings of this review emphasize the importance of increasing public awareness regarding the risks associated with the consumption of wild mushrooms. Educational campaigns improved toxicological monitoring, and early clinical recognition of mushroom poisoning are essential measures for reducing the incidence of severe intoxications and fatalities. In addition, further interdisciplinary research integrating mycology, toxicology, and epidemiology is needed to improve the identification of toxic species, understand regional risk patterns, and support the development of effective prevention strategies.

## Figures and Tables

**Table 1:** Major poisonous wild mushrooms and associated clinical syndromes (as reported in the reviewed literature.

Poisonous Species/Genus	Toxin Class (as Stated)	Predominant Clinical Syndrome	Geographic Notes	References
*A. phalloides*	Amatoxins/cyclopeptides (reported as leading lethal/cyclopeptide-related)	Severe intoxication; major contributor to fatalities	Europe; also principal lethal species in Europe and the Americas; major cause of deaths in France/Germany; primary culprit in Turkey	[5,6,7,8]
*A. exitialis*	Amatoxin-containing/cyclopeptide-related lethal poisoning (reported)	High lethality; acute liver failure prominent among fatal outcomes; multiple deaths reported	Southern China; leading cause of mortality among species (2019–2023)	[9,10,11,12,13,14]
*A. fuliginea*	Not specified (species-level attribution in fatal outcomes)	Major contributor to intoxications/deaths; fatal outcomes mainly via acute liver failure (overall system data)	China (China CDC system data)	[11]
*A. subjunquillea*	Not specified (species-level attribution)	Fatal mushroom poisoning (primary causes listed)	China	[9,12]
*A. pseudoporphyria*	Not specified (species-level attribution)	Fatal mushroom poisoning (primary causes listed)	China	[9,12]
*A. subpallidorosea*	Not specified (species-level attribution)	Fatal mushroom poisoning (primary causes listed)	China	[9,12]
*A. rimosa*	Not specified (species-level attribution)	Fatal mushroom poisoning (primary causes listed)	China	[9,12]
*A. proxima*	Not specified (species-level attribution in morbidity)	Moderate–severe intoxication reported in poison center data	Israel	[15]
*A. virosa*	Not specified (species-level attribution in mortality)	Reported mortality 1.55% in a hospital cohort	Western Iran (Kermanshah province)	[16]
*A. verna*/*A. virosa*	Not specified	Principal lethal species group (as stated)	Europe and the Americas	[17]
*Chlorophyllum molybdites*	Not specified	Frequently involved in non-fatal outbreaks; multiple incidents reported	China	[18,19]
*Russula subnigricans*	Not specified (species-level attribution)	Identified among species responsible for a substantial proportion of incidents in system analysis	China	[11]
*Russula* spp. (myotoxic cohort)	Not specified (species noted in myotoxic cohort)	Myotoxic mushroom intoxication cohort outcomes (hospitalizations; deaths reported for cohort)	Thailand	[20]
*Tricholoma ustale*	Ustalic acid referenced in methods paper; intoxication cases reported	Intoxication cases (case count reported)	Japan	[21]
*Gyromitra* spp.	Gyromitrin (stated)	Serious poisonings historically documented; toxin identification described	Europe and USA (historical/poison center trends summarized)	[22]
*Cortinarius* spp.	Nephrotoxic mushrooms (stated)	Nephrotoxic syndrome with acute renal failure; toxidromic approach described	Global review; commonly implicated	[2]
*A. smithiana*	Nephrotoxic mushrooms (stated)	Nephrotoxic syndrome (acute renal failure context)	USA/Canada	[2]
*Tricholoma equestre*	Nephrotoxic mushrooms (stated in nephrotoxic review context)	Nephrotoxic mushroom intoxication context	Europe	[2]
*A. muscaria*	Not specified	Intoxication cases including severe outcomes; distribution noted	Europe/UK distribution; cases reported in Turkey	[23,24]
*Gymnopilus junonius*	Psilocybin (stated)	Hallucinogenic intoxication potential	Worldwide distribution	[25]
*Inosperma muscarium*, *Inosperma hainanense*	Muscarine content (stated)	Muscarine-related toxicity potential (species described with “unexpected muscarine content”)	Tropical China	[26]

**Table 2:** Summary of epidemiological studies on wild mushroom poisoning and mortality worldwide.

Country/Region	Study Period	Poisonous Species (When Identified)	Main Findings/Outcomes	References
Romania & Eastern Europe (context)	Not specified	Not specified	Increased mushroom consumption/foraging linked to rising emergency presentations	[34]
France	Not specified (annual estimate)	*A. phalloides*	~1300 cases/year; deaths mainly attributed to *A. phalloides*	[5]
Türkiye	Not specified	*A. phalloides*	Identified as primary culprit; high toxin potency noted	[6]
Slovakia	2004–2020	*A. phalloides*	2876 poisonings; 698 suspected *A. phalloides*, 141 confirmed	[35]
Israel (Haifa Poison Information Center)	2015–2020 (focus on 2020)	*Lepiota brunneoincarnata*, *A. proxima*	105 calls in 2020; 61.90% in last quarter; moderate–severe intoxication in 6%	[15]
Israel (Rambam/Poison Information Center data)	2010–2021 (plus 2017–2021 detail)	Not specified	Wild mushrooms = 4% of biological agent exposures; males > females (*p* < 0.004); highest shares: >18 y (41%) and <6 y (39%); 128 raw-consumption cases (2017–2021), mainly <6 y	[31]
USA (HCUP database)	2016	Not specified	1328 ± 100 ED visits; 100 ± 22 hospitalizations; sex and age-group distribution reported	[36]
Türkiye (Elazig; pediatric)	January 2015–October 2017	Not specified	143 hospitalized children; wild mushroom poisoning in 7/19 toxic plant/mushroom cases (36.84%); more common in spring	[37]
Japan	1989–2010	*Tricholoma ustale*	86 cases affecting 347 patients	[21]
India (North-Eastern India)	January 2015–December 2020	Not specified	44 patients; age distribution reported	[38]
Iran (Mazandaran; Qaemshahr)	2015–2018	Not specified	65 hospitalized; spring peak (60%); seasonal distribution given	[39]
Iran (Kermanshah)	March 2014–March 2018	Not specified	193 patients; sex distribution; most aged 21–60	[16]
Iran (Kermanshah)	2018	Cyclopeptide-containing mushrooms	Outbreak with 283 patients; outpatient share ~43%; ~40% hospitalized 1–3 days; age distribution given	[40]
Thailand	2003–2017	Not specified	22,571 cases; strong rainfall correlation; seasonality; rural vs. urban difference	[41]
Thailand (Ramathibodi Poison Center surveillance)	January 2012–December 2016	*Russula* spp. (3 patients)	41 myotoxic cases; 70.73% hospitalized within 24 h; median time to admission 21 h	[20]
China	Up to end of 2022	Multiple; 196 species in incidents	196 species identified in incidents; 97 species causing six clinical types; 12 newly documented poisonous species	[9]
China (Xingtai City, Hebei)	2023	Amatoxin-containing wild mushrooms	Fatal intoxications posed significant threat in the region	[19]
China	2019	*Chlorophyllum molybdites*	55 food intoxication incidents due to accidental consumption	[18]
China (Chuxiong Yi Autonomous Prefecture People’s Hospital)	2019–2024	*A. exitialis*	10 events/27 individuals; clinical timing (latency, admission delay), hospital stay reported	[10]
China	2012–2023	*A. exitialis*, *Russula subnigricans* (noted as major)	Lab-confirmed species identification in 14.08% of incidents; *A. exitialis* and *R. subnigricans* account for 17.39% of toxic species cited	[11]
China	2010–2020	Multiple toxic mushrooms	10,036 outbreaks; 38,676 cases; 788 deaths; geographic distribution; household setting 84.6%; seasonal peak May–Oct	[42,43]
China (Zhejiang)	January 2016–December 2018	Not specified	429 cases from 340 sentinel hospitals; incidence rate 0.2526/100,000; hospitalizations by year reported	[42]
China	2022	Multiple (98 species; 7 clinical types)	482 incidents across 21 PLADs; 1332 patients; 98 species; 3 provisional new species + additional newly recorded species	[12]
China	2019	Multiple (≈70 toxic species; 6 syndromes)	276 incidents across 17 PLADs; 769 patients; monthly distribution with peak in July; market/dried/mixed exposures described	[44]
China	2021	Multiple (74 species; 6 syndromes)	327 investigations across 25 PLADs; 923 patients; 15 newly recorded species	[45]
China (Chuxiong Prefecture)	2015–2020	Species identified in 57.34% (unspecified in text)	4841 hospitalized in 17 hospitals; species info available for 2776 (57.34%)	[46]
China	2020	Wild mushrooms (as cause category)	2705 mushroom outbreaks among 4662 foodborne outbreaks; mushrooms = 58.02% of outbreaks; 9111 patients	[47]
Europe	Not specified (annual estimate)	Not specified	~50–100 fatal cases/year	[7]
Germany	2000–2018	*A. phalloides* (≈90% of lethal)	4412 hospitalizations; 22 deaths; ~90% lethal due to *A. phalloides*	[8]
Türkiye	January 2018–December 2023	Not specified	30,459 admissions; 30- and 90-day mortality 4.38% and 6.56%; deceased older (*p* = 0.001)	[6]
USA	January 2008–December 2018	Cyclopeptide-containing wild mushrooms (subset confirmed)	8953 exposures; 13 deaths among 148 patients (as reported); mortality comparisons with/without silibinin/silymarin	[49]
Iran (Kermanshah)	Not specified (within cohort described)	*A. virosa*	Mortality 1.55% (3 deaths among 193)	[16]
India (Meghalaya)	January 2015–December 2020	Not specified	10 deaths among 44 patients; in-hospital mortality 22.73%	[38]
Thailand	Not specified (reported as country estimate)	Not specified (amatoxin intoxication)	Amatoxin intoxication main cause of death; mortality rate 27.3%	[50]
Thailand	2003–2017	Not specified	106 deaths registered; temporal peak noted (e.g., 2012)	[41]
Thailand	January 2012–December 2016	Not specified (myotoxic; *Russula* noted earlier in same cohort context)	11 deaths among 41 (26.82%); demographics given	[20]
China	Not specified (species list + multiple time windows)	*A. exitialis*, *A. subjunquillea*, *A. pseudoporphyria*, *A. subpallidorosea*, *A. rimosa*	Species listed as primary causes; *A. exitialis* noted as most lethal	[9,12]
China	2019–2023	*A. exitialis*	135 cases; 24 deaths; leading cause of mortality among species	[9,12]
China (Yunnan)	2019	*A.exitialis*	1 death among children in family outbreak	[13]
China (Shenzhen)	April 2022	*A.exitialis*	1 death among 10 patients	[14]
China (Chuxiong hospital)	2019–2024	*A.exitialis*	5 deaths among 27 (18.52%)	[10]
China (China CDC PH Emergency Management Info System)	2012–2023	*A. exitialis*, *A. fuliginea*	Most lethal outcomes due to acute liver failure (55.17%); species shares for intoxications/deaths reported	[11]
China (Chuxiong Prefecture)	2015–2017 vs. 2018–2020	Not specified	Mean mortality decreased 0.57% → 0.06% (*p* < 0.001)	[46]
China	2010–2020 + 2020 detail	Not specified	10,036 outbreaks and 788 deaths; in 2020: 80 lethal cases; 55.94% of foodborne outbreak-related deaths; CFR 0.9%	[47,51,52]
China	2019/2021/2022/2023	Not specified	CFR: 2.86% (2019), 2.17% (2021), 2.10% (2022); 2023 investigation: 303 patients, 16 deaths, CFR 1.23%	[9,12,44,45]
China (Dalian hospital)	July 2007–August 2017	Not specified	66 patients; liver injury/failure distribution; 10/22 deaths in liver failure group (45.45%)	[53]
China (Zhejiang)	January 2016–December 2018	Not specified	2 deaths in 2016 among 429 patients; mortality 0.47%	[42]
India	Not specified	Not specified	3 patients died despite combined treatment	[54]
USA (Columbia)	Not specified	Presumed *Amanita*	1 death after family ingestion; death 4 days post-ingestion	[55]

## Data Availability

No new data were created or analyzed in this study. Data sharing is not applicable to this article.

## References

[1] Tang J.K.S., Phan C.W., Tan Y.S., Sabaratnam V., Seelan J.S.S., Bolhassan M.H. (2022). Bibliometric analysis of mushroom poisoning: From diversity to clinical management. Int. J. Med. Mushrooms.

[2] Diaz J.H. (2021). Nephrotoxic mushroom poisoning: Global epidemiology, clinical manifestations, and management. Wilderness Environ. Med..

[3] Codjia J.E.I., Sánchez-Ramírez S., Ndolo Ebika S.T., Wu G., Margaritescu S., Komura D.L., Oliveira J.J.S., Ryberg M., Tulloss R.E., Yorou N.S. (2023). Historical biogeography and diversification of ringless Amanita (section Vaginatae) support an African origin and suggest niche conservatism in the Americas. Mol. Phylogenet. Evol..

[4] He M.Q., Wang M.Q., Chen Z.H., Deng W.Q., Li T.H., Vizzini A., Jeewon R., Hyde K.D., Zhao R.L. (2022). Potential benefits and harms: A review of poisonous mushrooms in the world. Fungal Biol. Rev..

[5] Le Daré B., Ferron P.J., Gicquel T. (2021). Toxic effects of amanitins: Repurposing toxicities toward new therapeutics. Toxins.

[6] Turan Gökçe D., Arı D., Ata N., Gökcan H., İdilman R., Ülgü M.M., Harputluoglu M., Akarsu M., Karasu Z., Ayvalı M.O. (2024). Mushroom intoxication in Türkiye: A nationwide cohort study based on demographic trends, seasonal variations, and the impact of climate change on incidence. Turk. J. Gastroenterol..

[7] Stoeva-Grigorova S., Yarabanova I., Radeva-Ilieva M., Ivanova D., Zlateva S., Marinov P. (2025). Acute *Amanita muscaria* toxicity: A literature review and two case reports in elderly spouses following home preparation. Toxins.

[8] Wennig R., Eyer F., Schaper A., Zilker T., Andresen-Streichert H. (2020). Mushroom poisoning. Dtsch. Ärztebl. Int..

[9] Li H.J., Zhang Y.Z., Zhang H.S., Zhou J., Chen Z.H., Liang J.Q., Yin Y., He Q., Jiang S.F., Zhang Y.T. (2024). Mushroom poisoning outbreaks—China, 2023. China CDC Wkly..

[10] Chen C.G., Xu P., Li J.P., Bi X.L., Yao Q.M., Yu C.M., Tang Y., Sun C.Y., Wu Z.J., Zhong J.J. (2025). Comprehensive clinical profile of *Amanita exitialis* poisoning: Integrating toxin detection and autopsy pathology. Toxins.

[11] Yuan Y., Zhang Y., Zhou J., Lang N., Jiang S., Li H., He Q., Zhang Y., Cheng B., Zhong J. (2024). Detection and identification in reported mushroom poisoning incidents—China, 2012–2023. China CDC Wkly..

[12] Li H.J., Zhang Y.Z., Zhang H.S., Zhou J., Liang J.Q., Yin Y., He Q., Jiang S.F., Zhang Y.T., Yuan Y. (2023). Mushroom poisoning outbreaks—China, 2022. China CDC Wkly..

[13] Zhong J.J., Yao Q.M., Li H.J., Zhang Y.Z., Peng J.M., Yu C.M. (2023). *Amanita exitialis* poisoning in five patients. Clin. Toxicol..

[14] Meng H., Chen Z.Y., Chen L.C., Tang W.X., He F., Yan X.R., Lin X.H., Se X.L., Xie M.F., Li Z.H. (2023). An outbreak of *Amanita exitialis* poisoning. Clin. Toxicol..

[15] Lurie Y., Lewinsohn D., Kurnik D. (2022). An outbreak of mushroom poisoning in Israel during the 2020 fall and winter season: An unexpected outcome of COVID-19 restrictions?. Clin. Toxicol..

[16] Janatolmakan M., Ganji M.R., Ahmadi-Jouybari T., Rezaeian S., Ghowsi M., Khatony A. (2022). Demographic, clinical, and laboratory findings of mushroom-poisoned patients in Kermanshah province, west of Iran. BMC Pharmacol. Toxicol..

[17] Caré W., Bruneau C., Rapior S., Langrand J., Le Roux G., Vodovar D. (2024). Syndrome phalloïdien: Mise au point. Rev. Med. Interne.

[18] Wang N., Zhao Z., Gao J., Tian E., Yu W., Li H., Zhang J., Xie R., Zhao X., Chen A. (2021). Rapid and visual identification of Chlorophyllum molybdites with loop-mediated isothermal amplification method. Front. Microbiol..

[19] Lv B., Liu L., Xiao H., Meng Q., Zhang R., An Y., Jin Y., Ma Y., Gao H., Li Y. (2024). Food-borne poisoning accident from amanitin toxin in wild mushrooms—Xingtai City, Hebei province, China, 2023. China CDC Wkly..

[20] Trakulsrichai S., Jeeratheepatanont P., Sriapha C., Tongpoo A., Wananukul W. (2020). Myotoxic mushroom poisoning in Thailand: Clinical characteristics and outcomes. Int. J. Gen. Med..

[21] Yoshioka N., Hayakawa I., Minatani T., Tomozawa J., Akiyama H., Yomo H. (2020). Quantitative analysis of the *Tricholoma ustale*-derived toxin, ustalic acid, in mushroom and food samples by LC-MS/MS. Forensic Sci. Int..

[22] Horowitz K.M., Kong E.L., Regina A.C., Horowitz B.Z. (2025). *Gyromitra* mushroom toxicity. StatPearls (Internet).

[23] Rampolli F.I., Kamler P., Carnevale Carlino C., Bedussi F. (2021). The deceptive mushroom: Accidental *Amanita muscaria* poisoning. Eur. J. Case Rep. Intern. Med..

[24] Meisel E.M., Morgan B., Schwartz M., Kazzi Z., Cetin H., Sahin A. (2022). Two cases of severe *Amanita muscaria* poisoning including a fatality. Wilderness Environ. Med..

[25] Cho S.E., Jo J.W., Kwag Y.N., Lee H., Chung J.W., Oh S.H., Kim C.S. (2021). Complete mitochondrial genome sequence of *Gymnopilus junonius*. Mitochondrial DNA B Resour..

[26] Deng L.S., Kang R., Zeng N.K., Yu W.J., Chang C., Xu F., Deng W.Q., Qi L.L., Zhou Y.L., Fan Y.G. (2021). Two new *Inosperma* (Inocybaceae) species with unexpected muscarine contents from tropical China. MycoKeys.

[27] Zhang Y.Z., Yao Y., Zhang K.P., Liang J.Q., Zhong J.J., Li Z.F., Li H.J., Xu F. (2025). Toxin profiling of *Amanita citrina* and *A. sinocitrina*: First report of buiotenine detection. Toxins.

[28] Zhang S., Fan M., Zhang Y., Li S., Lu C., Zhou J., Zou L. (2024). Establishment and validation of a nomogram model for prediction of clinical outcomes in patients with *Amanita phalloides* poisoning. Heliyon.

[29] Zhang B., Liu Y., Li S., Li R., Zhang Y., Zhao H. (2025). Application of graphitized multi-walled carbon nanotubes combined with orbitrap high-resolution mass spectrometry for the rapid detection of ten toxins in wild mushrooms. Toxins.

[30] An X., Cheng G., Gao H., Yang Y., Li D., Li C., Li Y. (2022). First report of *Cladobotryum verticillatum* (Ascomycota, Hypocreaceae) causing cobweb disease on *Paxillusinvolutus*. Biodivers. Data J..

[31] Lewinsohn D., Lurie Y., Gaon A., Biketova A.Y., Bentur Y. (2023). The epidemiology of wild mushroom poisoning in Israel. Mycologia.

[32] Alvarado P., Gasch-Illescas A., Morel S., Dagher-Kharrat M.B., Moreno G., Manjón J.L., Carteret X., Bellanger J.M., Rapior S., Gelardi M. (2022). *Amanita* section *Phalloideae* species in the Mediterranean basin: Destroying angels reviewed. Biology.

[33] Tran H.H., Juergens A.L. (2025). Mushroom toxicity. StatPearls (Internet).

[34] Oprita B., Neacsu M.C., Dinu B.A., Olaru I., Oprita R. (2025). Wild/woodland mushroom poisoning: The experience of Bucharest emergency hospital—Retrospective study of ER 2023–2024 presentations. J. Fungi.

[35] Dluholucký S., Snitková M., Knapková M., Cibirová M., Mydlová Z. (2022). Results of diagnostics and treatment of Amanita phalloides poisoning in Slovakia (2004–2020). Toxicon.

[36] Gold J.A.W., Kiernan E., Yeh M., Jackson B.R., Benedict K. (2021). Health care utilization and outcomes associated with accidental poisonous mushroom ingestions—United States, 2016–2018. MMWR Morb. Mortal. Wkly. Rep..

[37] Gul E., Sari M.Y. (2020). Analysis of toxic plant and mushroom poisoning in children. Ann. Med. Res..

[38] Tiewsoh I., Bhattacharya P.K., Barman B., Barman H., Rapthap K., Sangla L., Lynrah K.G. (2022). Delayed liver toxicity and delayed gastroenteritis: A 5 year retrospective analysis of the cause of death in mushroom poisoning. J. Fam. Med. Prim. Care.

[39] Khatir I.G., Hosseininejad S.M., Ghasempouri S.K., Asadollahpoor A., Moradi S., Jahanian F. (2020). Demographic and epidemiologic evaluation of mushroom poisoning: A retrospective study in 4-year admissions of Razi Hospital (Qaemshahr, Mazandaran, Iran). Med. Glas..

[40] Karami Matin B., Amrollahi-Sharifabadi M., Rezaei S., Heidari A., Kazemi-Karyani A. (2022). Epidemiology and economic burden of an outbreak of cyclopeptide-containing mushroom poisoning in the West of Iran. Front. Public Health.

[41] Somrithipol S., Pinruan U., Sommai S., Khamsuntorn P., Luangsa-Ard J.J. (2022). Mushroom poisoning in Thailand between 2003 and 2017. Mycoscience.

[42] Chen L., Sun L., Zhang R., Liao N., Qi X., Chen J., Liu T. (2021). Epidemiological analysis of wild mushroom poisoning in Zhejiang province, China, 2016–2018. Food Sci. Nutr..

[43] Li W., Pires S.M., Liu Z., Liang J., Wang Y., Chen W., Liu C., Liu J., Han H., Fu P. (2021). Mushroom poisoning outbreaks—China, 2010–2020. China CDC Wkly..

[44] Li H., Zhang H., Zhang Y., Zhang K., Zhou J., Yin Y., Jiang S., Ma P., He Q., Zhang Y. (2020). Mushroom poisoning outbreaks—China, 2019. China CDC Wkly..

[45] Li H., Zhang H., Zhang Y., Zhou J., Yin Y., He Q., Jiang S., Ma P., Zhang Y., Yuan Y. (2022). Mushroom poisoning outbreaks—China, 2021. China CDC Wkly..

[46] Yao Q., Wu Z., Zhong J., Yu C., Li H., Hu Q., He J., Du J., Sun C. (2023). A network system for the prevention and treatment of mushroom poisoning in Chuxiong Autonomous Prefecture, Yunnan Province, China: Implementation and assessment. BMC Public Health.

[47] Li H., Li W., Dai Y., Jiang Y., Liang J., Wang S., Zhuang M., Huang Z., Xu L., Xue B. (2021). Characteristics of settings and etiologic agents of foodborne disease outbreaks—China, 2020. China CDC Wkly..

[48] Dogan M., Simseker O.F., Karayel F., Uzun I. (2025). Clinicopathological features of fatal mushroom poisoning: A 10-year retrospective autopsy-based study. Forensic Sci. Med. Pathol..

[49] De Olano J., Wang J.J., Villeneuve E., Gosselin S., Biary R., Su M.K., Hoffman R.S. (2021). Current fatality rate of suspected cyclopeptide mushroom poisoning in the United States. Clin. Toxicol..

[50] Jongthun R., Hemachudha P., Wacharapluesadee S., Hemachudha T. (2020). Low-cost management of mushroom poisoning in a limited-resource area: A 12-year retrospective study. Trop. Dr..

[51] He Z., Li X., Feng M., Wang X., Wang Y., Chen J. (2024). Wild mushroom poisoning: A case study of amatoxin-containing mushrooms and implications for public health. Toxicon.

[52] Su H., Jiang Z.-H., Hsu Y.-W., Wang Y.-C., Chen Y.-Y., Wu D.-C., Shiea J., Lee C.-W. (2024). Rapid identification of mushroom toxins by direct electrospray probe mass spectrometry for emergency care. Anal. Chim. Acta.

[53] Gao Y., Zhang H., Zhong H., Yang S., Wang Q. (2021). Lactate and blood ammonia on admission as biomarkers to predict the prognosis of patients with acute mushroom poisoning and liver failure: A retrospective study. Toxicol. Res..

[54] Vaibhav V., Meshram R., Yashpal S., Jha N., Khorwal G. (2023). Mushroom poisoning: A case series with a literature review of cases in the Indian subcontinent. Cureus.

[55] Connors N.J., Hoffman R.S., Gosselin S. (2020). Just the facts: Management of cyclopeptide mushroom ingestion. Can. J. Emerg. Med..

